# Association Between Gender Minority Status and Mental Health in High School Students

**DOI:** 10.1016/j.jadohealth.2022.12.028

**Published:** 2023-02-19

**Authors:** James White, Laurence Moore, Rebecca Cannings-John, Jemma Hawkins, Chris Bonell, Matthew Hickman, Stanley Zammit, Linda Adara

**Affiliations:** a Centre for Trials Research, School of Medicine, Cardiff University, Cardiff, UK; b MRC/CSO Social and Public Health Sciences Unit, Institute of Health and Wellbeing, University of Glasgow, Glasgow, UK; c DECIPHer, School of Social Sciences, Cardiff University, Cardiff, UK; d Department of Public Health, Environment and Society, London School of Hygiene & Tropical Medicine, London, UK; e Population Health Sciences, University of Bristol, Bristol, UK

**Keywords:** Gender minority, Depression, Anxiety, Conduct problem, Hallucination

## Abstract

**Purpose:**

Adolescence is a phase when young people begin to explore their gender identity. Adolescents who identify as a gender minority are vulnerable to experiencing mental health problems due to stigmatization of their identity.

**Methods:**

A population-wide study compared gender minority and cisgender students (aged 13–14 years) self-reported symptoms of probable depression, anxiety, and conduct disorder, and auditory hallucinations, including the distress and frequency of hallucinations.

**Results:**

Gender minority students compared to cisgender students had four times the odds of reporting a probable depressive disorder, anxiety disorder, auditory hallucinations, but not conduct disorder. Of those who reported a hallucination, gender minority students were more likely to report hearing them daily but were no more likely to find them distressing.

**Discussion:**

Gender minority students experience a disproportionate burden of mental health problems. Services and programming should be adapted to better support gender minority high-school students.

The prevalence of mental health problems among high school youth is a major public health issue. Around 10%–20% of all adolescents experience some type of mental health problem [1]. Gender identity in adolescence can be varied in its formation and expression. Students identifying as a gender minority (identity that differs from their assigned sex at birth or does not fit within the male-female categorization) are more likely to report bullying than their cisgender (identity that corresponds to sex assigned at birth) peers [2] and having to deal with the usual stressors of adolescence. There has been a growing policy focus on gender minority mental health [3], but there have been few population-based studies comparing gender minority to their cisgender peers [4].

Systematic reviews of gender minority patients identified from health records, sampled from gender minority clinics, or undergoing hormonal or surgical treatment related to their identity report that the prevalence of mental health disorders is higher than that reported in general population samples [5,6]. These studies have, however, involved small samples, and a lack of matched cisgender comparators, and very less focus on adolescents. One study with 12–29-year-olds in Boston (n = 360) found that transgender patients had a higher prevalence of diagnosed anxiety and depressive disorders than a matched cisgender population [7]. It is questionable although whether estimates gathered from gender minority patients are generalizable to the wider adolescent population.

In two randomly sampled population-level studies, gender minority college [8] and high school students [9] reported more depressive symptoms than cisgender students. Another U.S. populatione–based sample found high school students who identified as transgender, or questioned their gender identity, were more likely to report symptoms of hopelessness than cis-gender students [10]. However, other symptoms common to adolescence like anxiety and conduct problems [5,6] and those elevated in some other minority groups, such as psychosis [11], have not been investigated in adolescence. Accordingly, this study aimed to compare these four mental health outcomes between gender minority and cisgender high school students.

## Methods

Data were from baseline assessments within a cluster randomized controlled trial conducted in England and Wales (study protocol and eligibility criteria described elsewhere [12]). Of the 311 schools within the study areas, 244 (78.5%) met the eligibility criteria. A random sample of these eligible schools was invited, stratifying more and less than the median percentage of students entitled to free-school meals, an index of parental socioeconomic disadvantage. Questionnaires were completed by ear nine students (aged 13–14 years) in schools with the assistance of fieldworkers between September 2019 and March 2020. Data were collected before random allocation of schools. Parents/carers provided an informed opt out consent and students informed a written opt in consent. All procedures were approved by Cardiff University’s School of Social Sciences Ethics Committee (SREC/3342). This manuscript adheres to the STROBE reporting guidelines [13].

Probable depressive disorder was assessed using all questions from the 13-item Short Mood and Feelings Questionnaire applying the ≥ 12 cut-off point to indicate a disorder [14]. Probable anxiety disorders were assessed using all questions from the seven-item Generalized Anxiety Disorder scale applying the ≥ 10 cut-off point [15]. Probable conduct disorder was assessed using all questions from The Oregon Adolescent Depression Project Conduct Disorder Screener with the ≥ 9 cut-off [16]. Auditory hallucinations were self-reported using three questions from the World Health Organization Composite International Diagnostic Interview [17] (questions used listed in the Supplement). Young people who reported a hallucination were asked about the distress caused by this experience (response options ranging from not to very distressing) and frequency (not at all to nearly every day/daily).

Gender minority status was self-reported by students using the question, “Which of the following options best describes how you think of yourself? boy; girl; transboy; transgirl; nonbinary (neither male or female); unsure/questioning; other; or prefer not to say”. We categorized respondents as cisgender (boy/girl) or gender minority, excluding prefer not to say.

Covariates were sociodemographic factors including self-reported age, ethnicity, parental unemployment, free school meal entitlement, and substance use, comprising weekly smoking status (at least one cigarette a week), consumption of a whole alcoholic drink in the past 30 days, and lifetime illicit drug use.

Missing data per variable ranged from 1.0% to 27.7%. We imputed missing data using multiple imputations to generate 20 datasets. We estimated odds ratios for the association between gender minority status and outcomes using multilevel logistic regression (students nested within schools). These are reported alongside 95% confidence intervals. The association between gender-minority status and outcomes was adjusted for self-reported age, ethnicity, parental unemployment, and free school meal entitlement. Next, to examine potential mechanisms, we added the substance use variables. To reduce the risk of generating spurious findings due to multiple testing, the threshold for significance was Bonferroni adjusted to *p* < .004 (*p* = .05/12). Sensitivity analyses were conducted after excluding participants with any missing data.

## Results

Of 7,077 eligible students, 6,672 participated (94.3% response) which are our analytical sample. There were 6,522 (97.7%) students who identified as cisgender, 108 (1.6%) as gender minority, and 42 (0.6%) preferred not to say. Gender minority students were more likely to come from a socioeconomically deprived household and have used illicit drugs but were no more likely to have smoked or used alcohol (Table 1).

After adjusting for sociodemographic characteristics, gender minority students had four times the odds of cisgender students in reporting a probable depressive disorder, generalized anxiety disorder, and auditory hallucination (Table 2). Among students who reported a hallucination, gender-minority students were more likely to report hearing them every day but no more likely to be distressed. There was weaker evidence of a difference in conduct disorder. There was little attenuation in estimates after additional adjustment for substance use.

The results of sensitivity analysis in the 2,791 participants with no missing data were not materially different to those using the imputed sample (Table A1).

## Discussion

In this population-based study, gender-minority students were significantly more likely than their cisgender peers to report symptoms consistent with a probable depressive disorder, anxiety disorder, and auditory hallucinations, but not conduct disorder.

In agreement with results from the three previous studies which sampled adolescents from the general population, we found large inequalities between gender-minority and cisgender students reporting depressive symptoms [2,3,10]. In the New Zealand–based Youth’12 study of gender-minority students aged 13–18 years [9], and in the U.S. Healthy Minds Study of transgender and gender-queer U.S. college students, gender-minority students were 2–4 times more likely to screen for a probable depressive disorder [8]. In the 2017 Healthy Kids Colorado Survey [10], transgender students aged 14–18 years were also 2–4 times more likely to report hopelessness than cisgender heterosexual students.

We are not aware of other studies that have investigated symptoms of anxiety as per gender-minority status in high school students. Our findings are similar to those from one study of U.S. college students which found that transgender and gender queer students were more likely to screen for an anxiety disorder [8]. They also replicate those from a study using electronic health records which found transgender 12–29-year-olds were three times more likely to have anxiety disorder than a matched group of controls [7]. Our findings are novel in that they provide the first evidence that these differences are apparent in younger adolescents.

To our knowledge, our study is also the first to compare the experience of auditory hallucinations as per gender-minority status. Other minority groups, such as lesbian, gay and bisexual people [18], and ethnic minorities [11], report auditory verbal hallucinations more often than do majority-group peers, and psychotic experiences index the more severe end of psychopathology in young people [19]. This consistency in reporting of hallucinations across minority groups suggests similar mechanisms of marginalization and stigma may underpin these experiences.

Limitations include the small number of transgender, nonbinary, and questioning students, which prevented disaggregated analysis. It is possible that the prevalence of gender-minority students was underestimated as students who responded that they were a boy or girl may have had a different assigned sex at birth. We also did not ask whether students understood what gender identity meant. This could have led to underestimation and reduced power but is unlikely to have introduced systematic biases. These results were from 13–14-year-olds and they may not generalize to younger or older ages. Factors such as harassment and social support, that may help explain the differences we found, were also not assessed.

Our findings signal to educators and clinicians a need for policies, organizational practices, and programs to better support the mental health needs of gender minority high school students. Examples include Gay-Straight Alliances, where lesbian, gay, bisexual, transgender, and queer youth and their allies attempt to improve the school climate for sexual and gender minority youth [20], teacher and wider staff education to foster greater acceptance of gender minority group identification [21], and antibullying programs [22], which have shown some promise in reducing LGBTQ victimization.

## Supplementary Material

Supplementary data

## Figures and Tables

**Table 1:** Characteristics of young people by self-reported gender identity^a^

Characteristic	% (no.)		
	Cisgender (n = 6,522)	Gender minority (n =108)	*p* Value
Sociodemographic Characteristics			
Age (years) (Mean, SD)	13.69 (0.32)	13.72 (0.31)	.49
Ethnicity			
White British	83.4 (5,101)	76.8 (83)	
White not British	3.7 (437)	6.0 (6)	
Asian or Asian British	5.5 (222)	4.7 (8)	
Black or Black British	1.4 (137)	3.6 (2)	
Mixed/multiple ethnic backgrounds	4.4 (359)	4.0 (7)	
Other	1.6 (266)	4.9 (2)	.07
Entitled to free school meals	11.7 (763)	20.4 (22)	.006
Parent(s) unemployed	5.9 (385)	10.8 (12)	.05
Substance use			
Weekly smoker	1.5 (97)	2.4 (3)	.60
Alcohol consumed in past 30 days	19.9 (1,298)	20.3 (22)	.91
Lifetime illicit drug user	22.8 (1,487)	35.8 (39)	.001

a Cisgender comprises students identifying as a boy or girl. Gender minority comprises students who identified as a transboy, transgirl, nonbinary (neither male or female), unsure/questioning, and other.

**Table 2:** Self-reported probable depressive disorder, generalized anxiety disorder, conduct disorder, and auditory hallucinations by gender minority status

Outcome^a^	% (no.)		Odds ratio (95% confidence interval)			
	Cisgender (n = 6,522)	Gender minority (n = 108)	Adjusted for sociodemographic factors^a^	*p* value^c^	Adjusted for substance use^b^	*p* value^c^
Probable depressive disorder	22.0 (1,435)	57.3 (62)	4.85 (3.24, 7.24)	<.001	4.85 (3.21, 7.33)	<.001
Probable generalized anxiety disorder	19.3 (1,259)	52.7 (57)	4.73 (3.16, 7.07)	<.001	4.72 (3.13, 7.14)	<.001
Probable conduct disorder	22.0 (1,435)	32.8 (35)	1.58 (1.03, 2.44)	.03	1.44 (0.89, 2.33)	.14
Auditory hallucinations^d^	16.9 (1,102)	53.7 (58)	5.68 (3.49, 9.23)	<.001	5.22 (3.17, 8.62)	<.001
	(n = 796)	(n = 39)				
Sub-Group who reported an auditory hallucination						
Very distressed by hearing voices	12.0 (96)	20.1 (8)	1.79 (0.68, 4.70)	.38	1.56 (0.57, 4.26)	.38
Hearing voices nearly every day or daily	14.8 (118)	37.0 (14)	3.28 (1.55, 6.91)	.002	3.35 (1.58, 7.10)	.002

a Adjusted for age, ethnicity, parental unemployment, free school meal entitlement.

b Adjusted for age, ethnicity, parental unemployment, free school meal entitlement, plus smoking status, alcohol consumption, and illicit drug use.

c Statistically significant differences in the regression models defined as a Bonferroni-corrected *p* < .004 for 12 comparisons.

d Analytical n = 4,791 (cisgender, n = 4,719; minority, n = 72) as excludes students who responded that they preferred not to say or did not know whether they had hallucinated.
