# Supplementary material for: Association Between Gender Minority Status and Mental Health in High School Students
Source: J Adolesc Health. Author manuscript; Available in PMC 2025 Feb 24. (PMC10824667; doi:10.1016/j.jadohealth.2022.12.028)
Supplement: Supplementary data [file EMS193566-supplement-Supplementary_data.zip › 1-s2.0-S1054139X23000198-mmc2.docx]

**eTable 1. Odds ratio (95 % confidence interval) for association between gender minority status with probable depressive, generalized anxiety, and conduct disorder, auditory hallucinations in sample with no missing data (n = 2791)**

| **Outcome** **^a^** | **Adjusted for sociodemographic factors ^a^** | ***P* Value** | **Adjusted for substance use variables ^b^** | ***P* Value** |
| --- | --- | --- | --- | --- |
|  |  |  |  |  |
| Probable depressive disorder | 9.32 (4.63, 18.74) | <0.001 | 7.99 (3.86, 16.53) | <0.001 |
| Probable generalized anxiety disorder | 10.80 (5.38, 21.69) | <0.001 | 9.37 (4.56, 19.27) | <0.001 |
| Probable conduct disorder | 1.96 (0.98, 3.92) | 0.06 | 1.28 (0.59, 2.81) | 0.53 |
| Auditory hallucinations | 7.76 (3.94, 15.29) | <0.001 | 6.45 (3.17, 13.13) | <0.001 |
|  |  |  |  |  |
| **Sub-Group who reported an auditory hallucination** | |  |  |  |
| Very distressed by hearing voices | 2.71 (0.81, 9.03) | 0.11 | 2.34 (0.68, 8.01) | 0.18 |
| Hearing voices nearly every day or daily | 3.00 (1.04, 8.69) | 0.04 | 3.05 (1.04, 8.96) | 0.04 |

^a^ Adjusted for age, ethnicity, parental unemployment, free school meal entitlement.

^b^ Adjusted for age, ethnicity, parental unemployment, free school meal entitlement, plus smoking status, alcohol consumption, and illicit drug use.

^c^ Analytical n = 347 (cisgender, n = 329; minority, n = 18) as excludes students who responded that they preferred not to say or didn’t know whether they had hallucinated.

**The World Health Organization Composite International Diagnostic Interview questions**

1. Did you ever hear things that other people said did not exist, like strange voices coming

from inside your head talking to you or about you, or voices coming out of the air when

there was no one around? (Please do not include any times when you were dreaming or

half-asleep) (Please mark an **X** in **ONE** box only)

Yes No Prefer not to say Don’t know

**2**. How distressing did you find this experience?

(Please mark an **X** in **ONE** box only)

**3.** How often did this experience happen in the **past year**? (Please mark an **X** in **ONE** box only)

Not at all

Once or twice

Less than once a month

More than once a month

Nearly every day or daily

Prefer not to answer

Not distressing – it was a positive experience

Not distressing – it was a neutral experience

A bit distressing

Quite distressing

Very distressing

Don’t know

Prefer not to answer
